# Effect of climate warming on the annual terrestrial net ecosystem CO_2_ exchange globally in the boreal and temperate regions

**DOI:** 10.1038/s41598-017-03386-5

**Published:** 2017-06-08

**Authors:** Zhiyuan Zhang, Renduo Zhang, Alessandro Cescatti, Georg Wohlfahrt, Nina Buchmann, Juan Zhu, Guanhong Chen, Fernando Moyano, Jukka Pumpanen, Takashi Hirano, Kentaro Takagi, Lutz Merbold

**Affiliations:** 1 School of Environmental Science and Engineering, Guangdong Provincial Key Laboratory of Environmental Pollution Control and Remediation Technology, Sun Yat-sen University, Guangzhou, 510275 China; 2 Directorate for Sustainable Resources, European Commission, Joint Research Centre, Ispra, I-21027 Italy; 3 Institute of Ecology, University of Innsbruck, Sternwartestr 15, Innsbruck, 6020 Austria; 4 Institute of Agricultural Sciences, ETH Zürich, Universitaetsstrasse 2, Zürich, 8092 Switzerland; 5 Department of Bioclimatology, Georg-August University of Göttingen, Büsgenweg 2, Göttingen, 37077 Germany; 6 Department of Environmental and Biological Sciences, University of Eastern Finland, Kuopio, 70211 Finland; 7 Research Faculty of Agriculture, Hokkaido University, Sapporo, 060-8589 Japan; 8 Northern Forestry and Development Office, Field Science Center for Northern Biosphere, Hokkaido University, Horonobe, 098-2943 Japan

## Abstract

The net ecosystem CO_2_ exchange is the result of the imbalance between the assimilation process (gross primary production, GPP) and ecosystem respiration (RE). The aim of this study was to investigate temperature sensitivities of these processes and the effect of climate warming on the annual terrestrial net ecosystem CO_2_ exchange globally in the boreal and temperate regions. A database of 403 site-years of ecosystem flux data at 101 sites in the world was collected and analyzed. Temperature sensitivities of rates of RE and GPP were quantified with *Q*_10_, defined as the increase of RE (or GPP) rates with a temperature rise of 10 °C. Results showed that on the annual time scale, the intrinsic temperature sensitivity of GPP (*Q*_10*sG*_) was higher than or equivalent to the intrinsic temperature sensitivity of RE (*Q*_10*sR*_). *Q*_10*sG*_ was negatively correlated to the mean annual temperature (MAT), whereas *Q*_10*sR*_ was independent of MAT. The analysis of the current temperature sensitivities and net ecosystem production suggested that temperature rise might enhance the CO_2_ sink of terrestrial ecosystems both in the boreal and temperate regions. In addition, ecosystems in these regions with different plant functional types should sequester more CO_2_ with climate warming.

## Introduction

The ecosystem CO_2_ exchange is controlled by fluxes associated with assimilation and respiration processes, corresponding to the rates of gross primary production (GPP) and ecosystem respiration (RE), respectively^1^. The net CO_2_ exchange (NEE) of terrestrial ecosystems, resulting from a delicate balance between GPP and RE, influences carbon dynamics and budget and is vulnerable to climate disturbance^2, 3^. However, there is still no consensus over the magnitude and directions of the response to climate change of the physiological processes underpinning the ecosystem CO_2_ exchange^4^. This uncertainty often limits the ability to predict the carbon balance of terrestrial ecosystems^5^.

Both GPP and RE are sensitive to temperature changes^6, 7^. Future variations in temperature may therefore modify the carbon sequestration capacity of ecosystems by altering the balance between plant growth and soil carbon decomposition^8^. As a consequence, global warming may have important impacts on the CO_2_ balance at all levels of ecological organization, from individual organisms to entire ecosystems^9^. For this reason, it is of great importance to investigate and link biotic responses to climate drivers and carbon cycle processes^10^.

The effect of temperature on the ecological respiration processes has been commonly quantified using the temperature sensitivity index *Q*_10_, which is defined as the increase of CO_2_ respiration rate with a temperature rise of 10 °C^11^. *Q*_10_ values fitted to measured field data with statistical models are normally used to characterize the temperature sensitivity of ecological respiration process. This observed temperature sensitivity is the outcome of many interacting processes and is referred to apparent sensitivity to differentiate it from the intrinsic temperature sensitivity, which reflects the direct effect of temperature on the kinetics of chemical reactions^12^. The temperature sensitivities of respiration are different across biomes and temporal scales^13^. It has been reported that apparent *Q*_10_ values of RE change with geographic locations, ecosystem types, and other factors^14^. Zhou *et al*.^15^ showed that apparent *Q*_10_ values of RE ranged from 1.43 to 2.03 in different biomes. Jones *et al*.^16^ found that intrinsic *Q*_10_ values of RE are in the range of 1.9 ± 0.4 to 2.1 ± 0.7 in the region from 5°N to 5°S. Using the FLUXNET database collected in the mid-latitude region of the northern hemisphere, Mahecha *et al*.^17^ obtained intrinsic *Q*_10_ of RE close to a constant of 1.4 ± 0.1. Wang *et al*.^18^ showed that intrinsic *Q*_10_ of RE ranged between 1.4 and 1.6 from more than 300 field measurements in the region from 19°N to 74°N.

The *Q*_10_ approach is on the base of the relationship between reaction rates and temperature described by van’t Hoff^19^. As shown above, this exponential model has been widely used to study temperature sensitivity of RE at the subcellular and individual levels, as well as at the ecosystem level^13, 17, 20, 21^, including calculations of apparent and intrinsic temperature sensitivity indexes of RE. The relationship holds over the temperature range of normal activity, which can be up to 40 °C for most organisms^22^. Similar to respiration, assimilation is also a temperature related reaction process. Therefore, it is expected that the exponential model should be applicable to quantify the temperature sensitivity of CO_2_ assimilation rate. Applications of the same type of models to quantify the temperature effects on RE and GPP can make it easier to compare the difference of temperature sensitivities between RE and GPP. Nevertheless, applicability of the exponential model to temperature sensitivity of GPP needs to be further demonstrated.

With limited studies, some have shown that RE is more sensitive than GPP to climate warming^23, 24^. In contrast, others have reported that GPP was more sensitive than RE to climate warming^25, 26^. Lupascu *et al*.^27^ reported a permafrost ecosystem as a strong carbon sink, whereas Xue *et al*.^28^ showed a significant net carbon loss in permafrost-based tundra with climate warming. Some others have observed a simultaneous greening trend globally with climate warming^29–31^. The findings reported in the literature show that there is no consensus and that we still have no clear understanding of temperature sensitivities of the assimilatory and respiratory processes as well as their influence on the annual terrestrial NEE. We used extensive data to investigate the temperature dependency of the CO_2_ balance processes in terrestrial ecosystems at the global scale. Specifically, the objectives of this study were to examine intrinsic temperature sensitivities of the assimilatory and respiratory processes in terrestrial ecosystems of the boreal and temperate regions, in ecosystems featuring different plant functional types (PFTs), and to estimate the response of annual terrestrial NEE to climate warming.

## Results

**Figure 1:**
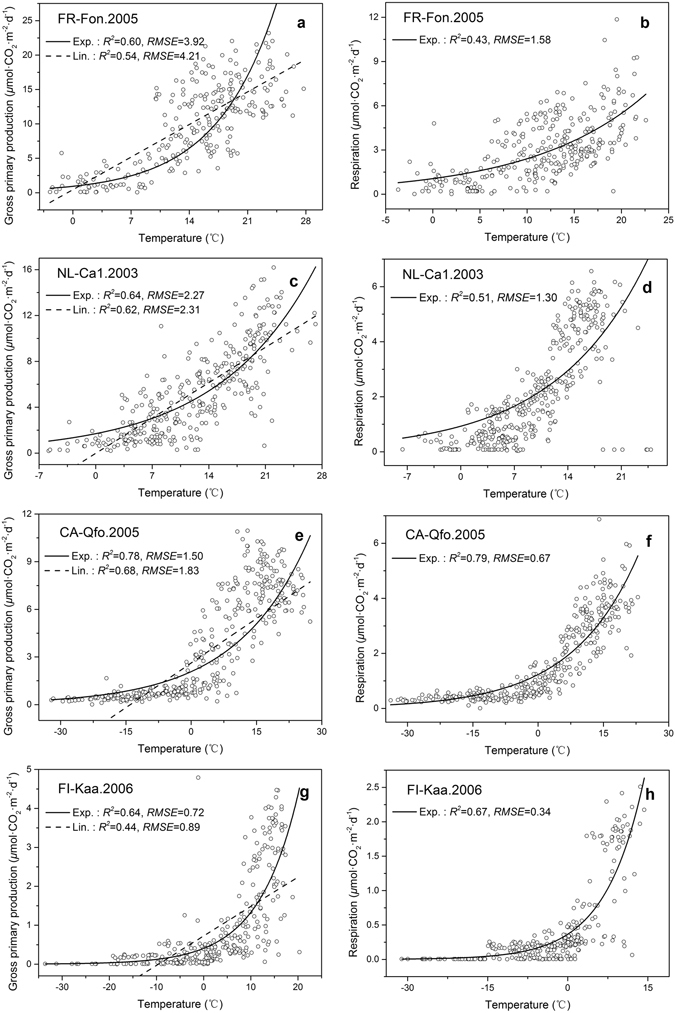
Fitting results using different models to gross primary production rates (GPP) and respiration rate (RE) vs. temperature. The exponential model (i.e., the *Q*_10_ approach, Exp.) and the linear function model (Lin.) were used to gross primary production rates (GPP) vs. temperature at site-years of (**a**) FR-Fon.2005, (**c**) NL-Ca1.2003, (**e**) CA-Qfo.2005, and (**g**) FI-Kaa.2006, and the exponential model was used to fit respiration rates (RE) vs. temperature at site-years of (**b**) FR-Fon.2005, (**d**) NL-Ca1.2003, (**f**) CA-Qfo.2005, and (**h**) FI-Kaa.2006. Sites of FR-Fon, NL-Ca1, CA-Qfo, and FI-Kaa represent ecosystems featuring with climate regions (i.e., temperate and boreal) and plant functional types (PFTs) (i.e., forest and non-forest) of temperate & forest, temperate & non-forest, boreal & forest, and boreal & non-forest, respectively. Comparison of the fitting results was based on the coefficients of determination (*R* ^*2*^) and root mean square error (*RMSE*).

To demonstrate the applicability of the exponential model to temperature sensitivity, GPP data collected at the 403 site-years (see Supplementary Table 1) were fitted with the exponential model (Eq. 2, see the section of Methods) and a linear model. The measured RE data were also fitted with the exponential model. The fitting results of GPP and RE were compared using values of the coefficients of determination (*R* ^*2*^) and of the root mean square error (RMSE). The results of the comparison demonstrates that temperature sensitivities of both RE and GPP can be well characterized by the exponential model. As examples, fitting results of GPP and RE for some site-years, which represent ecosystems featuring with climate regions and PFT groups of temperate & forest, temperate & non-forest, boreal & forest, and boreal & non-forest, respectively, are shown in Fig. 1. Then the approximate intrinsic temperature sensitivity indexes of GPP (*Q*_10*sG*_) and RE (*Q*_10*sR*_) were determined on all site-years using the procedure of Mahecha *et al*.^17^ described in the section of Methods.

### Temperature sensitivity indexes in the whole region

**Figure 2:**
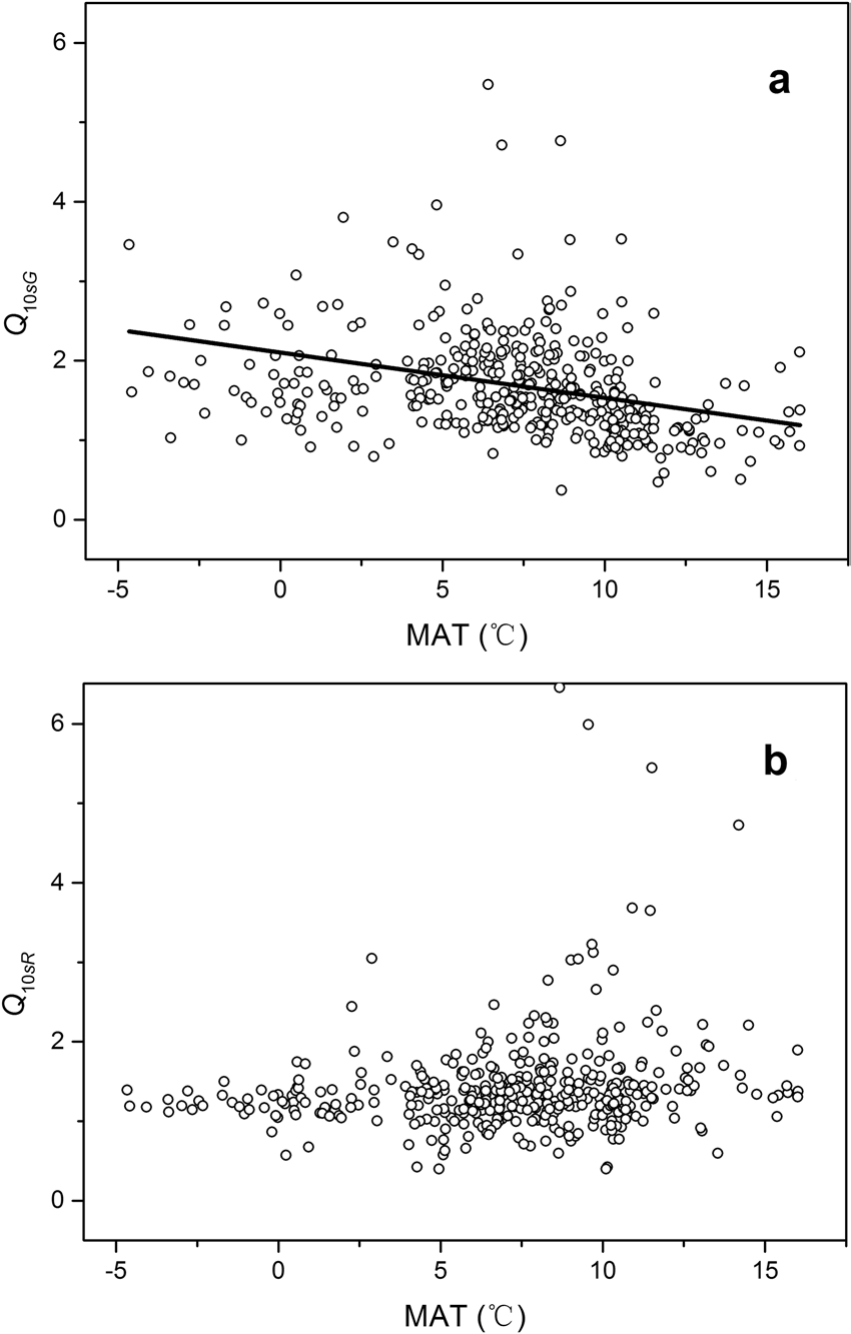
Relationships between intrinsic temperature sensitivity indexes of (**a**) gross primary production rate (GPP, *Q*_10*sG*_) and (**b**) respiration rate (RE, *Q*_10*sR*_) vs. mean annual temperature (MAT) (GPP: *Pearson’s r* = −0.31, *n* = 403, *F* = 41.80, *P* < 0.0001; RE: *r* = −0.034, *n* = 403, *F* = 2.65, *P* = 0.49). The solid line in (**a**) represents the linear regression line.

Considering the boreal and temperate areas as a whole region, the mean of *Q*_10*sR*_ was 1.43 ± 0.03 with the 95% confidence interval (95% CI) spanning from 1.37 to 1.50. The mean of *Q*_10*sG*_ was 1.69 ± 0.04 with the 95% CI from 1.62 to 1.76. The Wilcoxon rank-sum test showed that differences between *Q*_10*sR*_ and *Q*_10*sG*_ (Δ*Q*_10*s*_ = *Q*_10*sG*_ − *Q*_10*sR*_) among different sites were significant (*two*-*tailed test*, *Z* = 7.39, *P* < 0.0001), and generally GPP was more sensitive to temperature than RE. Values of *Q*_10*sG*_ were negatively correlated to the mean annual temperature (MAT) (*Pearson’s r* = −0.31, *linear regression*: *F* = −41.8, *P* < 0.0001), whereas *Q*_10*sR*_ values was not correlated to the MAT (*r* = −0.034, *F* = 2.65, *P* = 0.49) (Fig. 2).

### Temperature sensitivity indexes between different climate regions and plant functional types

**Figure 3:**
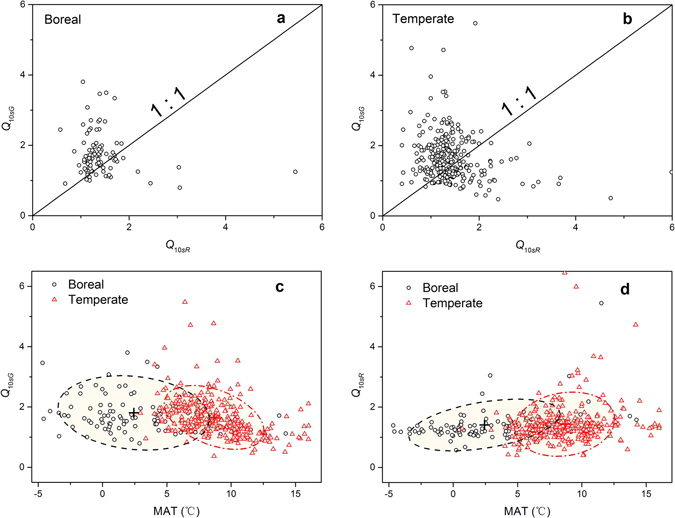
The intrinsic temperature sensitivity of gross primary production rate (*Q*_10*sG*_) vs. that of respiration rate (*Q*_10*sR*_) in (**a**) the boreal region and (**b**) the temperate region, and changes of (**c**) *Q*_10*sG*_ and (**d**) *Q*_10*sR*_ with the mean annual temperature (MAT) in the two regions. The confidence ellipses in (**c**,**d**) were derived at the level of one standard deviation of probability distribution and the symbol “+” indicates the center of ellipse.

In the boreal region, the mean values of *Q*_10*sR*_ and *Q*_10*sG*_ were 1.41 and 1.81, respectively. In the temperate region, the mean values of *Q*_10*sR*_ and *Q*_10*sG*_ were 1.43 and 1.66, respectively (Table 1). Values of *Q*_10*sG*_ were significantly different to those of *Q*_10*sR*_ within the climate regions (boreal: *Z* = 5.44, *P* < 0.0001; temperate: *Z* = 5.56, *P* < 0.0001) (Fig. 3a,b). However, the temperature sensitivities were not significantly different between the climate regions for both GPP (*Z* = 1.78, *P* = 0.075) and RE (*Z* = −0.19, *P* = 0.85), also indicated by the similar values of the centers of confidence ellipses at the level of one standard deviation of probability distribution in Fig. 3c,d, respectively.

**Table 1:** Basic information, temperature sensitivity indexes of assimilation rate (GPP, *Q*_10*sG*_) and respiration rate (RE, *Q*_10*sR*_), and net ecosystem productivity (NEP, *μ*mol · m^−2^ · yr^−1^) of different climate groups.

Climate group	MAT (°C)	Sample size	*Q*_10*s*_	Mean	Standard deviation	Confidence interval (95%)	Δ*Q*_10*s*_	NEP_0_	NEP trend
Boreal	2.43	93	*Q*_10*sG*_	1.81	0.08	(1.66, 1.98)	>0 (<0.0001)	=0 (0.13)	sink
			*Q*_10*sR*_	1.41	0.06	(1.30, 1.53)			
Temperate	8.67	310	*Q*_10*sG*_	1.66	0.04	(1.58, 1.74)	>0 (<0.0001)	>0 (<0.0001)	sink
			*Q*_10*sR*_	1.43	0.04	(1.36, 1.52)			

MAT is the mean annual temperature. Δ*Q*_10*s*_ = *Q*_10*sG*_ − *Q*_10*sR*_. NEP_0_ is the current net ecosystem productivity. Values in parentheses of the Δ*Q*_10*s*_ and NEP_0_ column are *P* results of the null hypothesis test: *P* < 0.05 indicates that Δ*Q*_10*s*_ or NEP_0_ is﻿ significantly different from zero. The NEP trends indicate that the ecosystem should be a CO_2_ sink or a CO_2_ source to the atmosphere with temperature rise.

**Figure 4:**
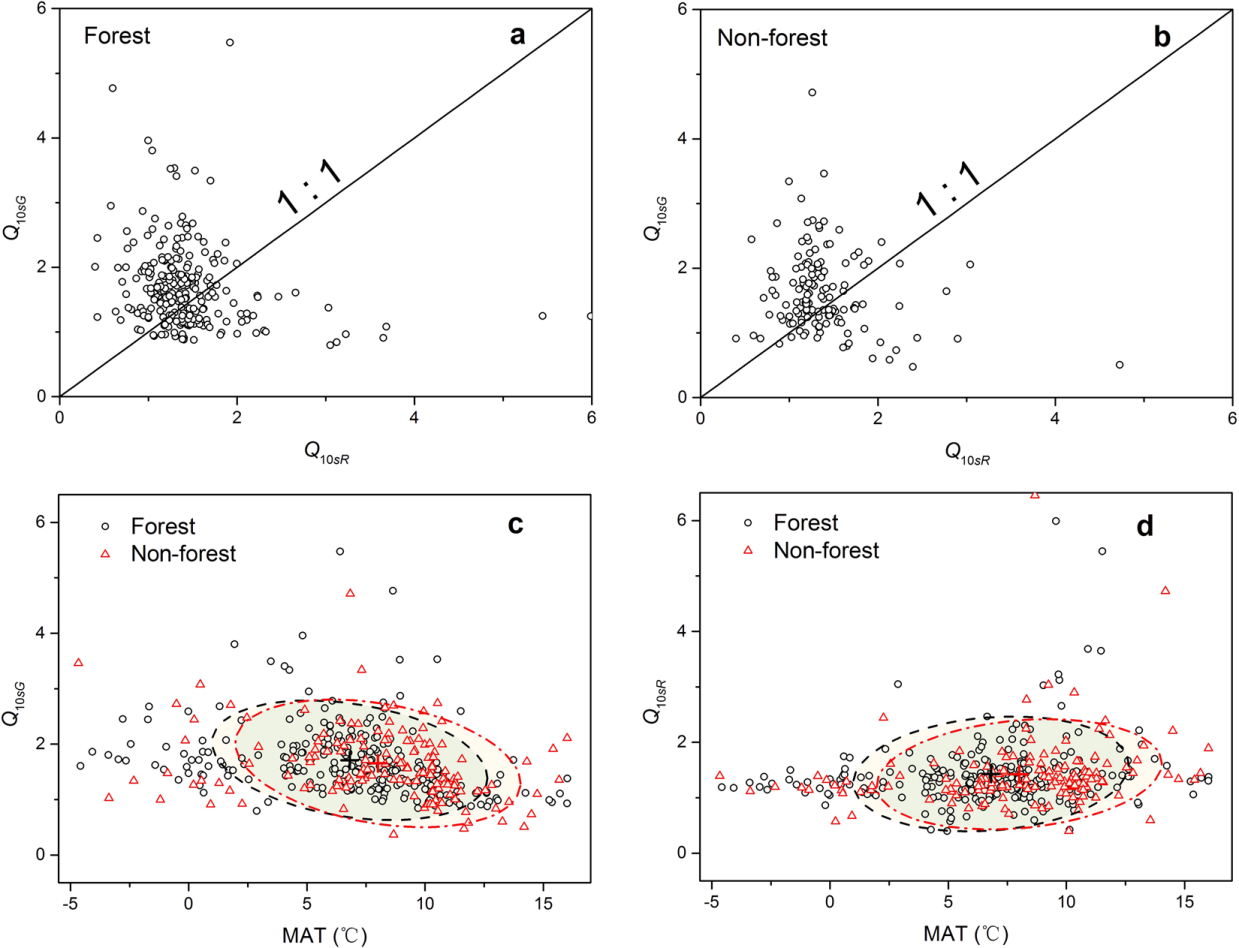
The intrinsic temperature sensitivity of gross primary production rate (*Q*_10*sG*_) vs. that of respiration rate (*Q*_10*sR*_) in ecosystems with plant function types in (**a**) the forest site group and (**b**) the non-forest site group, and changes of (**c**) *Q*_10*sG*_ and (**d**) *Q*_10*sR*_ with the mean annual temperature (MAT) in the two plant groups. The forest site group includes deciduous broadleaf forest, evergreen needleleaf forest, and mixed forest. The non-forest site group includes croplands, closed shrublands, grasslands, open shrublands, and permanent wetland. The confidence ellipses in (**c**,**d**) were derived at the level of one standard deviation of probability distribution and the symbol “+” indicates the center of ellipse.

In the forest sites, including deciduous broadleaf forest, evergreen needleleaf forest, and mixed forest, the mean values of *Q*_10*sR*_ and *Q*_10*sG*_ were 1.43 and 1.71, respectively. In the not-forest sites, including croplands, closed shrublands, grasslands, open shrublands, and permanent wetland, the mean values of *Q*_10*sR*_ and *Q*_10*sG*_ were 1.42 and 1.65, respectively (Table 2). Within a PFT group, the difference between *Q*_10*sG*_ and *Q*_10*sR*_ was significant (forest: *Z* = 6.56, *P* < 0.0001; non-forest: *Z* = 3.67, *P* = 0.0002) (Fig. 4a,b). However, the PFT groups did not significantly affect the temperature sensitivities of GPP (*Z* = −0.91, *P* = 0.36) and RE (*Z* = −0.13, *P* = 0.90), also shown by the similar values of the centers of confidence ellipses at the level of one standard deviation of probability distribution in Fig. 4c,d, respectively.

**Table 2:** Basic information, temperature sensitivity indexes of assimilation rate (GPP, *Q*_10*sG*_) and respiration rate (RE, *Q*_10*sR*_), and net ecosystem productivity (NEP, *μ*mol · m^−2^ · yr^−1^) of different plant functional types (PFTs).

PFTs	MAT (°C)	Sample size	*Q*_10*s*_	Mean	Standard deviation	Confidence interval (95%)	Δ*Q*_10*s*_	NEP_0_	NEP trend
Forest group	6.81	260	*Q*_10*sG*_	1.71	0.04	(1.63, 1.80)	>0 (<0.0001)	>0 (<0.0001)	sink
			*Q*_10*sR*_	1.43	0.04	(1.35, 1.52)			
Non-forest group	8.00	143	*Q*_10*sG*_	1.65	0.06	(1.53, 1.79)	>0 (<0.0001)	>0 (0.0001)	sink
			*Q*_10*sR*_	1.42	0.05	(1.33, 1.54)			

Plant functional types in the forest group include deciduous broadleaf forests, evergreen needleleaf forests, and mixed forests. The plant functional types in the non-forest group include croplands, closed shrublands, grasslands, open shrublands, and permanent wetlands. MAT is the mean annual temperature. NEP_0_ is the current net ecosystem productivity. Δ*Q*_10*s*_ = *Q*_10*sG*_ − *Q*_10*sR*_. Values in parentheses of the Δ*Q*_10*s*_ and NEP_0_ column are *P* results of the null hypothesis test: *P* < 0.05 indicates that Δ*Q*_10*s*_ or NEP_0_ is significantly different from zero. The NEP trends indicate that the ecosystem should be a CO_2_ sink or a CO_2_ source to the atmosphere with temperature rise.

### The annual net ecosystem CO_2_ exchange

Using values of *Q*_10*sG*_ and *Q*_10*sR*_ as well as current net ecosystem production (*NEP*_0_), we determined the sign of the annual NEP (i.e., >0 for CO_2_ sink and <0 for CO_2_ source) in terrestrial ecosystems under a scenario of climate warming (Δ*T* > 0). According to Eq. (9) (see the section of Methods), if *GPP*_0_ ≧ *RE*_0_ (i.e., *NEP*_0_ ≧ 0) and *Q*_10*sG*_ ≧ *Q*_10*sR*_ (i.e., Δ*Q*_10*s*_ ≧ 0), it derives that Δ*NEP* ≧ 0, indicating that the ecosystem should be a CO_2_ sink (or carbon neutral as Δ*NEP* = 0). If *NEP*_0_ < 0 and Δ*Q*_10*s*_ < 0, it derives Δ*NEP* < 0, indicating that the ecosystem should be a CO_2_ source to the atmosphere. If *NEP*_0_ < 0 and Δ*Q*_10*s*_ > 0 (or *NEP*_0_ > 0 and Δ*Q*_10*s*_ < 0), the NEE is dependent on the values of *NEP*_0_, Δ*Q*_10*s*_, and Δ*T*.

In the boreal region, the null hypothesis test showed that the current NEP (i.e., *NEP*_0_ in Eq. (9)) ≈0 (*Signed rank test*: *S* = 397.5, *P* = 0.13) (Table 1). Using Eq. (9) with *NEP*_0_ = 0 and Δ*Q*_10*s*_ > 0 (*S* = 1352, *P* < 0.0001) (Table 1), it derived that Δ*NEP* > 0, indicating that ecosystems in the region will response to a mean temperature increase by moving from the current state of near carbon neutral to a carbon sink. Moreover, in the temperate region, *NEP*_0_ > 0 (*S* = 19716, *P* < 0.0001) and Δ*Q*_10*s*_ > 0 (*S* = 9168, *P* < 0.0001), thus Δ*NEP* > 0 (Table 1). Ecosystems in the temperate region should therefore remain to act as carbon sinks, in response to a mean temperature increase.

In ecosystems with PFTs in the forest group, the CO_2_ budget was determined by GPP > RE (*NEP*_*0*_ > 0: *S* = 12513, *P* < 0.0001) and *Q*_10*sG*_ > *Q*_10*sR*_ (*S* = 7601, *P* < 0.0001), thus we obtained Δ*NEP* > 0, indicating that the ecosystems will be carbon sinks with climate warming. In the same way, for ecosystems with PFTs in the non-forest group, NEP (*NEP*_0_) > 0 (*S* = 3507, *P* < 0.0001) and Δ*Q*_10*s*_ > 0 (*S* = 2046, *P* < 0.0001), temperature rise should also lead to carbon sinks (Table 2).

More generally, the response of NEP (or NEE) to a change in mean air temperature can also be determined using Eq. (7) (see the section of Methods). If GPP ≥ RE, we have

1 $$\frac{dNEP}{dT}\ge \frac{GPP}{10}\,\mathrm{ln}\,\frac{{Q}_{10sG}}{{Q}_{10sR}}$$

If *Q*_10*sG*_ > *Q*_10*sR*_, the system will be a carbon sink. If GPP = RE and *Q*_10*sG*_ = *Q*_10*sR*_, the system will be carbon neutral. If GPP < RE and *Q*_10*sG*_ < *Q*_10*sR*_, the system will be a carbon source. This equation is useful to predict the future direction of change of the NEE in the various regions.

## Discussion

In this study, we used the SCAPE method^17^ to derive the intrinsic temperature sensitivity (*Q*_10*s*_), which relied on the high frequency fluctuations of measured time series and represent the short-term response of ecological processes to temperature change. The method reduces or excludes the influences of confounding factors (e.g., seasonal variability and water limitation)^1, 17, 32^, which should result in robust estimates of *Q*_10*sG*_ and *Q*_10*sR*_.

GPP principally occurs through the photosynthesis, an endothermal reaction process, which requires distinctive activation energies for different ambient temperatures. More activation energy is required to complete the same reaction at a lower temperature^33–35^. Gillooly *et al*.^20^ have shown that *Q*_10_ is positively correlated to activation energy. Therefore, the relationship between the temperature sensitivity of GPP and MAT (Table 1 and Fig. 2a) is attributable to the activation energy required in the different temperature zones.

On the contrary, respiration is a heat-releasing process that mainly depends on the availability of substrate (i.e., carbon sources). Because of the heterogeneity of carbon sources, it is expected that diverse activation energies and turnover times are required for decomposition of the different carbon pools in the ecosystem^12, 36^. The turnover time ranges from less than 1 yr for labile carbon pool to greater than 10^6^ yr for recalcitrant carbon pool^37, 38^. As mentioned above, the *Q*_10*sR*_ reflected the temperature response of short-term decomposition of labile carbon pool. The required activation energy for decomposition of the labile carbon pool is considered to be a constant^12, 20, 36^. As a consequence, the temperature sensitivity of RE (Table 1 and Fig. 2b) was not significantly correlated to temperature, consistent with the result of Mahecha *et al*.^17^.

At the ecosystem level, the changes of GPP and RE with temperatures are different^2, 39^. Within the range of low temperatures in the boreal and temperate regions, GPP increases with temperature faster than RE, which results in an increase of CO_2_ uptake under climate warming scenarios^40^.

The integrated response of an ecosystem may demonstrate the thermal optimality under temperature change and be interpreted as photosynthesis and respiration acclimation to temperature^41, 42^. The temperature acclimation process, also described as temperature adaption, indicates that with increasing global temperature, plants and microorganisms may generate reversible changes in a way that can optimize their functions under the warmer environment^43, 44^. Such adaptation mechanism should result in compensative responses of the ecological processes of GPP and RE for a change in temperature^45–47^. The changes of NEP response to temperature can be modulated by the disproportional extent of temperature acclimation of either GPP or RE^48^. It has been well documented that at the plant level, electron transport capacity and/or heat stability of Rubisco are enhanced with increasing temperature, which result in higher photosynthetic assimilation rates^42^. Furthermore, the increased CO_2_ uptake in warmer temperatures may also be contributed by extended growing seasons, increased nitrogen mineralization and root growth^29, 49^. Respiration generally increases with temperature, however, water stress and respiratory acclimation can offset or reverse the direct temperature effect^50, 51^.

Our results showed that ecosystems in the boreal and temperate regions with different PFTs would become carbon sink with climate warming. Evidences have shown that the Earth is becoming greener since 1980s, indicating an increase in GPP^52^. This increase in GPP is mainly attributable to the rising atmospheric CO_2_ level and temperature^30, 31, 53^. The potential greenhouse effect may further accelerate CO_2_ uptake of plants^54^. In fact, the climate regions and PFTs interactively affect the temperature sensitivities of CO_2_ balance processes^55, 56^. For instance, the needleleaf biome generally distributes in cold climates of the world^57^. Consequently, the temperature sensitivity results derived from the PFTs are generally consistent with those from the climate groups.

**Figure 5:**
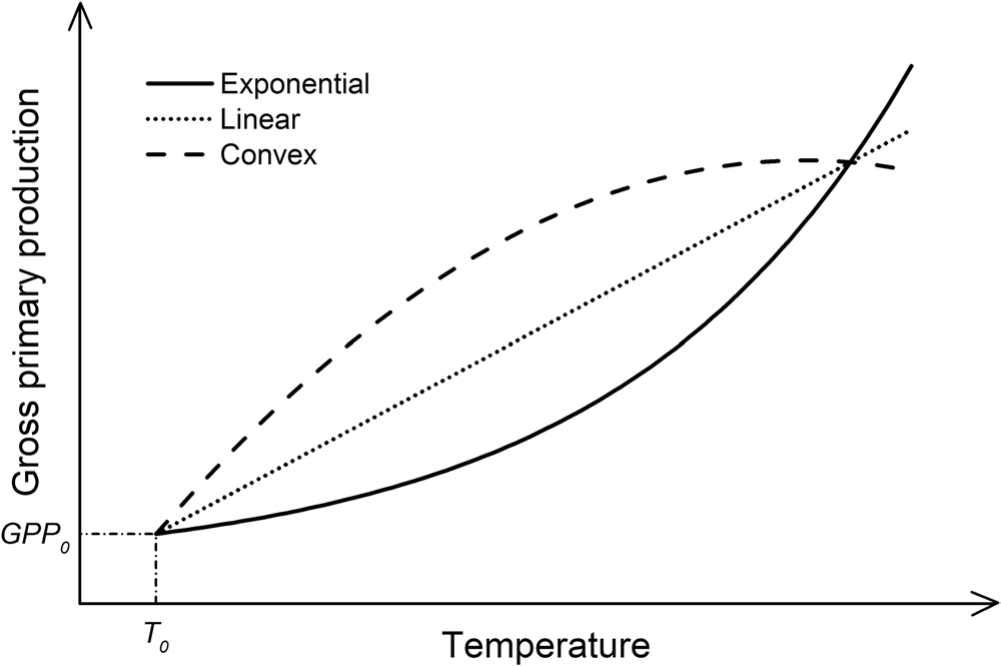
Conceptual models of the gross primary production (GPP) rates vs. temperature.

Besides the exponential function model (the *Q*_10_ approach) used in this study, other models, such as linear function^6^ and convex curve models^40^, have been applied to characterize GPP changes vs. temperature. It is well documented that GPP is positively correlated to temperature, but very high temperatures should decrease CO_2_ assimilation rates^58, 59^. Therefore, any model to characterize the GPP vs. temperature relationship should be applicable only within the temperature range for normal plant growth. Within the temperature range of this study (MAT: −4.67~16.0 °C), conceptually the exponential function model and the convex curve model should cross at a certain high temperature and the linear function model should be between the exponential function and the convex curve models. These models should cross at least at one point at the reference temperature (*GPP*_0_) (Fig. 5). Among the exponential function, linear function, and convex curve models, the exponential function model predicts the lowest GPP value for a given temperature. Thus, the NEP values calculated in this study are more conservative than those calculated using other models. In other words, the conclusion of NEE with “sink” for most of the cases in this study should hold when other models of GPP vs. temperature are applied.

It should be noted that the estimation of CO_2_ balance trends in this study was according to the GPP and RE results under a relatively short time scale. The GPP results were on an annual basis and the RE results were mainly related to the labile carbon pool with short turnover time in the ecosystem. The patterns of temperature sensitivities of GPP and RE, as well as the NEP changing with the climate regions and PFTs, provide valuable information to predict the CO_2_ balance processes in response to climate change at the global scale^60, 61^. However, in longer time scales, the effects of environmental constraints should become more profound, which may reduce sensitivities of the CO_2_ balance processes of terrestrial ecosystems to future climate warming^12, 60^. On the other hand, the wide range of kinetic properties demonstrate a diverse organic matter compounds of substrates and the recalcitrant organic carbon pool with longer turnover time is more sensitive to temperature than the labile organic carbon pool^36^. The long-term change of temperature sensitivities of GPP and RE should influence the responses and feedback of terrestrial ecosystem carbon dynamic to global temperature change. Therefore, further research is needed to explore the patterns of CO_2_ balance processes under long time scales.

## Methods

### Database

Time series of GPP and RE were assembled from the FLUXNET Database (http://www.fluxdata.org/, Open Data set), European Flux Database (European Fluxes Database Cluster http://gaia.agraria.unitus.it/), AmeriFlux (http://ameriflux.lbl.gov/), Distributed Active Archive Center for Biogeochemical Dynamics (ORNL DAAC http://daac.ornl.gov/). In total, 403 site-years at 101 sites throughout the world were collected. The datasets covered various ecosystems spanning between 40°N to 71°N. Except twelve site-years from Russia, other site-years were primarily from Europe and North America during the time period from 1991 to 2006 (see Supplementary Table S1).

Semi-continuous observations of NEE were obtained with the eddy covariance technique on a large number of experimental sites and ecosystem types^47^. All flux data were 30 min measured averages. Flux data sets were compiled according to site, year, ecosystem and vegetation type, environmental and physical variables, such as air temperature, latitude and longitude, precipitation, solar radiation, and NEE. The data were quality controlled following the procedures of Papale *et al*.^62^. The data were further interpolated (gap-filled) with the common procedures of the marginal distribution sampling method^63^ and the artificial neural network simulator^64^. The interpolation procedure of iterative singular spectrum analysis (SSA) was further used to reduce uncertainty^65^.

Daily ecosystem respiration rates were estimated from the mean NEE values measured during night^17^. At least five data points for each day and at least 255 measurements in each site were required for the estimations^17^. Mean air temperatures over the same periods for CO_2_ flux measurements were used as the corresponding temperatures for *Q*_10_ calculations. For forest sites, GPP values were estimated with the methods of using exclusive daytime fluxes^66^. For other sites, GPP values were derived from nighttime fluxes^63^. Since the absolute values of NEE and NEP are the same, daily NEP values were determined directly from the daily NEE measurements.

Our database included two climate regions, that is, the boreal region (between 49 and 71°N) (93 site-years) and the temperate region (between 40 and 64°N) (310 site-years). The collected data were also classified in the different PFTs according to the International Geosphere-Biosphere Programme (IGBP, 2012, http://www.igbp.net)^67^. In this study, we divided the database into two groups based on the PFTs, i.e., the forest site group (310 site-years) and the non-forest site group (143 site-years). The forest site group included deciduous broadleaf forest, evergreen needleleaf forest, and mixed forest. The not-forest site group included croplands, closed shrublands, grasslands, open shrublands, and permanent wetland.

### Calculation of *Q*_10_ values of GPP and RE

The relationship between reaction rates and temperature can be described by^19^:

2 $$R={R}_{r}{{Q}_{10}}^{\frac{T-{T}_{r}}{10}}$$

here *T* is the temperature (°C), *T*_*r*_ is the reference temperature, *R* and *R*_*r*_ are the measured reaction rates corresponding to *T* and *T*_*r*_, respectively (g C m^−2^ d^−1^), and *Q*_10_ is the temperature sensitivity index of the reaction. Equation (2) can be linearized as follows:

3 $$\mathrm{ln}\,R=\,\mathrm{ln}\,{R}_{r}+\frac{T-{T}_{r}}{10}\,\mathrm{ln}\,{Q}_{10}$$

By fitting Eq. (3) with measured *R* values at different temperatures, the apparent *Q*_10_ can be estimated by linear regression.

To study the annual ecosystem processes response to temperature changes, we are specifically interested in the sensitivity of these processes to fast temperature fluctuations. The SSA procedure was applied to extract the fast temperature fluctuations of the ecosystem processes directly from measured data^68, 69^. According to the procedure, a time series of measured data can be decomposed into sums of sub-signals of characteristic frequency ranges and overall mean values as follows^17^:

4 $$R(i)=\sum _{m=1}^{M}R{(i)}_{fm}+\langle R\rangle \quad i=1,2,\ldots ,N$$

5 $$T(i)=\sum _{m=1}^{M}T{(i)}_{fm}+\langle T\rangle \quad i=1,2,\ldots ,N$$

here *R*(*i*) is the series of reaction rates, *T*(*i*) is the corresponding temperature time series, the subscript *fm* represent the *m*th frequency band, *M* is the total number of predefined frequency bands, which should cover the whole frequency range, *N* is the time series length, and 〈 〉 denotes the overall time series mean. According to the scale parameter estimation (SCAPE) principle, different sub-signals are associated with parameters corresponding to different time scales. Reaction rates at high frequency bands reflect the fast temperature fluctuations, i.e., the direct responses to temperature, which is defined as the intrinsic temperature sensitivity^17^. The use of reaction rates and corresponding temperatures at high frequency bands should disentangle the effect of temperature on GPP and RE from all the other environmental factors. Therefore, the intrinsic temperature sensitivity can be related to the high frequency portions of *R*(*i*) and *T*(*i*) by ref. 17

6 $$\mathrm{ln}\,R{(i)}_{fh}=\frac{T{(i)}_{fh}-{T}_{r}}{10}\,\mathrm{ln}\,{Q}_{10s}$$

where the subscript *fh* represents the high frequency series and *Q*_10*s*_ is the intrinsic temperature sensitivity index. With measurement series of GPP and RE rates at different temperatures, the approximate intrinsic temperature sensitivity indexes of GPP (*Q*_10*sG*_) and RE (*Q*_10*sR*_) can be determined using the procedure of Mahecha *et al*.^17^ on the basis of Eqs (4) to (6).

The annual net CO_2_ exchange (i.e., the CO_2_ source or sink) of terrestrial ecosystems at increasing temperature can be defined as the sensitivity of NEP to temperature, which is related to the intrinsic temperature sensitivity indexes of GPP and RE as follows:

7 $$\frac{dNEP}{dT}=\frac{d(GPP-RE)}{dT}=\frac{GPP}{10}\,\mathrm{ln}\,{Q}_{10sG}-\frac{RE}{10}\,\mathrm{ln}\,{Q}_{10sR}$$

More specifically, at an initial condition (*T* = *T*_0_) and temperature rise to *T*_1_, the NEP values are calculated, respectively, with the following forms:

8a $$NE{P}_{0}=GP{P}_{0}-R{E}_{0}=GP{P}_{r}{Q}_{10sG}^{\frac{{T}_{0}-{T}_{r}}{10}}-R{E}_{r}{Q}_{10sR}^{\frac{{T}_{0}-{T}_{r}}{10}}$$

8b $$NE{P}_{1}=GP{P}_{1}-R{E}_{1}=GP{P}_{r}{Q}_{10sG}^{\frac{{T}_{1}-{T}_{r}}{10}}-R{E}_{r}{Q}_{10sR}^{\frac{{T}_{1}-{T}_{r}}{10}}$$

where *GPP*_*r*_ and *RE*_*r*_ are the GPP and RE values at the reference temperature, respectively. Therefore, with a temperature increase Δ*T* = *T*_1_ − *T*_0_, the change of NEP is:

9 $$\begin{array}{rcl}{\rm{\Delta }}NEP & = & NE{P}_{1}-NE{P}_{0}=GP{P}_{r}({Q}_{10sG}^{\frac{{T}_{1}-{T}_{r}}{10}}-{Q}_{10sG}^{\frac{{T}_{0}-{T}_{r}}{10}})-R{E}_{r}({Q}_{10sR}^{\frac{{T}_{1}-{T}_{r}}{10}}-{Q}_{10sR}^{\frac{{T}_{0}-{T}_{r}}{10}})\\  & = & GP{P}_{0}({Q}_{10sG}^{\frac{{\rm{\Delta }}T}{10}}-1)-R{E}_{0}({Q}_{10sR}^{\frac{{\rm{\Delta }}T}{10}}-1)\\  & = & GP{P}_{0}[{({Q}_{10sR}+{\rm{\Delta }}{Q}_{10s})}^{\frac{{\rm{\Delta }}T}{10}}-1)-R{E}_{0}({Q}_{10sR}^{\frac{{\rm{\Delta }}T}{10}}-1)\end{array}$$

where Δ*Q*_10*s*_ = *Q*_10*sG*_ − *Q*_10*sR*_.

### Data analysis

The uncertainty of mean *Q*_10_ values and confidence intervals were evaluated with the nonparametric bootstrap method^70^. This method is more advantageous than the normal distribution method to deal with uncertain population distributions with comparably small sample sizes. The bootstrap sample size was set as 10000, which is an appropriate value to ensure the reliability of results.

Both parametric and non-parametric tests were applied to examine the differences of *Q*_10_ among factors. Other statistical methods used for data analyses, included the null hypothesis test, the Wilcoxon rank-sum test, and the Kruskal-Wallis test^71, 72^. The SAS 9.3 software was used for the above analyses.

## Electronic supplementary material

Supplementary Information
