# Supplementary material for: Effect of climate warming on the annual terrestrial net ecosystem CO2 exchange globally in the boreal and temperate regions
Source: Sci Rep. 2017 Jun 8;7:3108. doi: 10.1038/s41598-017-03386-5 (PMC5465071; doi:10.1038/s41598-017-03386-5)
Supplement: Supplementary file 1 — Supplementary Information [file 41598_2017_3386_MOESM1_ESM.pdf]

Supplementary Information (SI) Appendix for

Effect of climate warming on the annual terrestrial net ecosystem CO_2 exchange globally in the boreal and temperate regions

Zhiyuan Zhang[1], Renduo Zhang[1,*], Alessandro Cescatti[2], Georg Wohlfahrt[3], Nina Buchmann[4], Juan Zhu[1], Guanhong Chen[1], Fernando Moyano[5], Jukka Pumpanen[6], Takashi Hirano[7], Kentaro Takagi[8] & Lutz Merbold[4]

[1]*School of Environmental Science and Engineering, Guangdong Provincial Key Laboratory of Environmental Pollution Control and Remediation Technology, Sun Yat-sen University, Guangzhou 510275, China*

[2]*Institute for Environment and Sustainability, Climate Risk Management Unit, European Commission, Joint Research Centre, Ispra I-21027, Italy*

[3]*Institute of Ecology, University of Innsbruck, Sternwartestr 15, Innsbruck 6020, Austria*

[4]*Institute of Agricultural Sciences, ETH Zürich, Universitaetsstrasse 2, Zürich 8092, Switzerland*

[5]*Department of Bioclimatology, Georg-August University of Göttingen, Büsgenweg 2, Göttingen 37077, Germany*

[6]*Department of Environmental and Biological Sciences, University of Eastern Finland, Kuopio 70211, Finland*

[7]*Research Faculty of Agriculture, Hokkaido University, Sapporo 060-8589, Japan*

[8]*Northern Forestry and Development Office, Field Science Center for Northern*

Biosphere, Hokkaido University, Horonobe 098-2943, Japan

* Corresponding author: Tel.: +86 20 84110052; fax: +86 20 84110692;

E-mail: zhangrd@mail.sysu.edu.cn

Materials and Methods

Data

Data sources of GPP and RE were assembled from the FLUXNET Database (http://www.fluxdata.org/, Open Data set), European Flux Database (European Fluxes Database Cluster http://gaia.agraria.unitus.it/), AmeriFlux (http://ameriflux.lbl.gov/), Distributed Active Archive Center for Biogeochemical Dynamics (ORNL DAAC http://daac.ornl.gov/). In total, 403 site-years at 101 sites were collected. The datasets covered various ecosystems between 40 °N to 71 °N, except 3 datasets from Russia, all data were primarily in Europe and North America and during the time period from 1991 to 2006 (Fig. S1). Our database included two main climate group, 93 of which were from the boreal zone (between 49 and 71 °N), and 310 of which were from temperate zone (between 40 and 64 °N). The collected data were classified by plant functional types (PFTs) according to the International Geosphere-Biosphere Programme (IGBP, 2012, http://www.igbp.net). In this study, data were divided into two major parts, 260 of which were from forest sites including deciduous broadleaf forest (DBF, *n*=65), evergreen needleleaf forest (ENF, *n*=165) and mixed forest (MF, *n*=30), 143 of which were from non-forest sites including croplands (CRO, *n*=40), closed shrublands (CSH, *n*=5), grasslands (GRA, *n*=61), open shrublands (OSH, *n*=13) and permanent wetland (WET, *n*=24). Basic information of the database, including site ID, latitude, longitude, PFT, climate group, year of data collection, and reference of each data set, is listed Table S1.

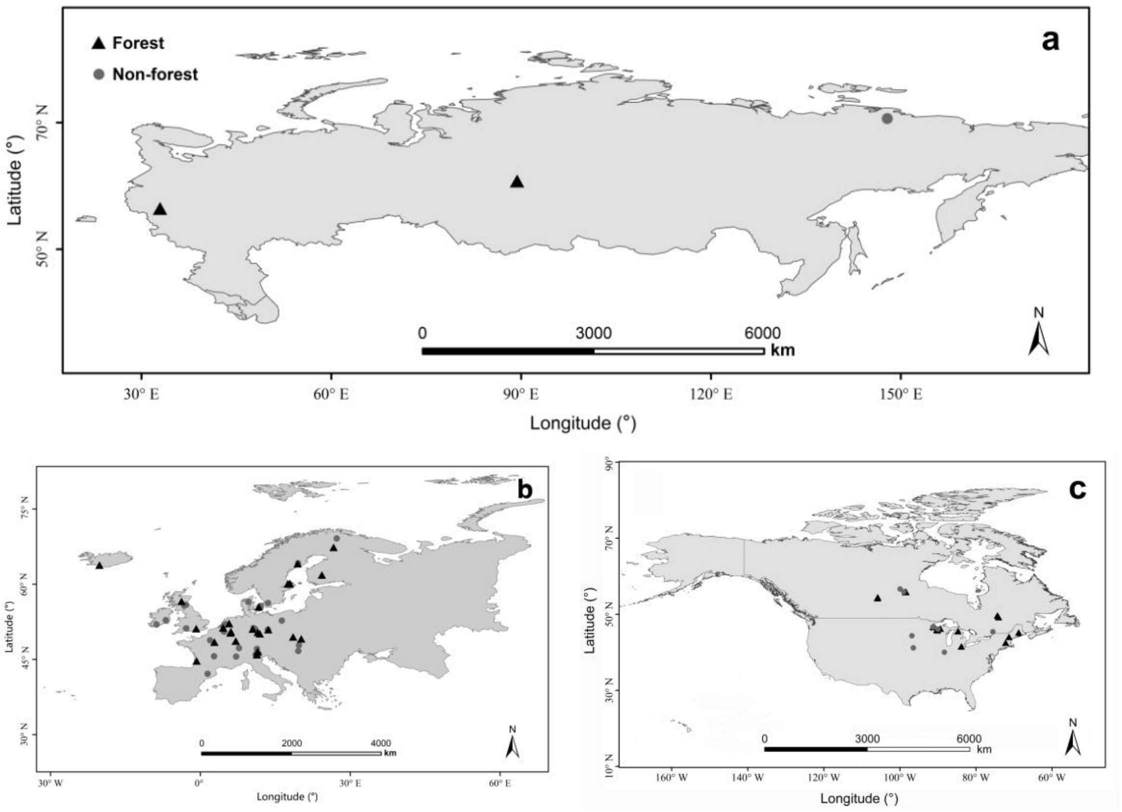

Fig. S1 Distribution of geographic locations of the collected sites in (a) Russia, (b) Europe, and (c) North American. Site information (i.e., latitude, longitude, and plant functional types) was collected from http://fluxnet.fluxdata.org/. The maps were created with ArcGIS 10.1 (ESRI Inc., USA, http://www.esri.com/arcgis/about-arcgis).

Table S1 Basic information (including site ID, latitude, longitude, plant functional type (PFT), climate group, year of data collection, and reference) of the database used in this study. All the information gathered from http://fluxnet.fluxdata.org/.

Site ID	Latitude[1]	Longitude[2]	PFT	Climate group	Year	Reference
AT-Neu	47.12	11.32	GRA	Temperate	2002-2004	1
BE-Bra	51.31	4.52	MF	Temperate	1997-1998, 2000-2002, 2004-2006	2
BE-Jal	50.56	6.07	MF	Temperate	2006	----
BE-Lon	50.55	4.74	CRO	Temperate	2004-2006	3
BE-Vie	50.31	6.00	MF	Temperate	1996-2006	4
CA-Man	55.88	-98.48	ENF	Boreal	1994-1995, 1997-2003	5
CA-Mer	45.41	-75.52	WET	Temperate-Continental with hot or warm summers	1999-2005	6
CA-NS1	55.88	-98.48	ENF	Boreal	2002-2005	7
CA-NS2	55.91	-98.52	ENF	Boreal	2001-2005	7
CA-NS3	55.91	-98.38	ENF	Boreal	2001-2005	7

CA-NS4	55.91	-98.38	ENF	Boreal	2003-2004	7
CA-NS5	55.86	-98.49	ENF	Boreal	2001-2005	7
CA-NS6	55.92	-98.96	OSH	Boreal	2001-2005	7
CA-NS7	56.64	-99.95	OSH	Boreal	2002-2005	6
CA-Qcu	49.27	-74.04	ENF	Boreal	2001-2006	8
CA-Qfo	49.69	-74.34	ENF	Boreal	2003-2006	9
CA-SF1	54.49	-105.82	ENF	Boreal	2003-2005	10
CA-SF2	54.25	-105.88	ENF	Boreal	2003-2005	10
CH-Oe1	47.29	7.73	GRA	Temperate	2002-2006	11
CH-Oe2	47.29	7.73	CRO	Temperate	2005	11
CZ-BK1	49.50	18.54	ENF	Temperate-Continental with hot or warm summers	2000-2006	12
DE-Bay	50.14	11.87	ENF	Temperate	1996-1999	13
DE-Geb	51.10	10.91	CRO	Temperate	2004-2006	14

DE-Gri	50.95	13.51	GRA	Temperate	2005-2006	15
DE-Hai	51.08	10.45	DBF	Temperate	2000-2006	16
DE-Kli	50.89	13.52	CRO	Temperate	2004-2006	17
DE-Meh	51.27	10.65	GRA	Temperate	2003-2006	18
DE-Tha	50.96	13.57	ENF	Temperate	1996-2006	19
DE-Wet	50.45	11.46	ENF	Temperate	2002-2006	20
DK-Fou	56.48	9.59	CRO	Temperate	2005	----
DK-Lva	55.68	12.08	GRA	Temperate	2005-2006	15
DK-Ris	55.53	12.10	CRO	Temperate	2004-2005	17
DK-Sor	55.49	11.65	DBF	Temperate	1996-2006	21
ES-VDA	42.15	1.45	GRA	Temperate	2004-2006	22
FI-Hyy	61.85	24.29	ENF	Boreal	1996-2006	23
FI-Kaa	69.14	27.30	WET	Boreal	2000-2006	----

FI-Sod	67.36	26.64	ENF	Boreal	2000-2006	23
FR-Fon	48.48	2.78	DBF	Temperate	2005-2006	22
FR-Gri	48.84	1.95	CRO	Temperate	2005-2006	22
FR-Hes	48.67	7.06	DBF	Temperate	1997-2006	24
FR-LBr	44.72	-0.77	ENF	Temperate	1996-1998,2000,2003-2005	25
FR-Lq1	45.64	2.74	GRA	Temperate	2004-2006	15
FR-Lq2	45.64	2.74	GRA	Temperate	2004-2006	15
HU-Bug	46.69	19.60	GRA	Temperate	2002-2006	----
HU-Mat	47.85	19.73	GRA	Temperate	2004-2006	26
IE-Ca1	52.86	-6.92	CRO	Temperate	2004-2006	15
IE-Dri	51.99	-8.75	GRA	Temperate	2003-2005	27
IS-Gun	63.83	-20.22	DBF	Temperate	1996-1998	----
IT-Lav	45.96	11.28	ENF	Temperate	2000-2002,2004,2006	28

IT-LMa	45.58	7.15	GRA	Temperate	2003-2006	----
IT-Mal	46.12	11.70	GRA	Temperate	2003-2006	15
IT-MBo	46.02	11.05	GRA	Temperate	2003-2006	29
IT-Ren	46.59	11.43	ENF	Temperate	1999-2006	30
NL-Ca1	51.97	4.93	GRA	Temperate	2003-2006	15
NL-Haa	52.00	4.81	GRA	Temperate	2003-2004	----
NL-Hor	52.03	5.07	GRA	Temperate	2004-2006	31
NL-Lan	51.95	4.90	CRO	Temperate	2005-2006	----
NL-Loo	52.17	5.74	ENF	Temperate	1996-2006	32
NL-Mol	51.65	4.64	CRO	Temperate	2005-2006	----
PL-wet	52.76	16.31	WET	Temperate	2004-2005	33
RU-Cok	70.62	147.88	OSH	Boreal	2003-2005	----
RU-Fyo	56.46	32.92	ENF	Temperate-Continental with hot or warm summers	1998-2004	34

RU-Zot	60.80	89.35	ENF	Boreal	2002-2004	----
SE-Deg	64.18	19.55	WET	Boreal	2001-2005	35
SE-Faj	56.27	13.55	WET	Temperate	2005-2006	36
SE-Fla	64.11	19.46	ENF	Boreal	1996-1998,2001-2002	37
SE-Nor	60.09	17.48	ENF	Temperate-Continental with hot or warm summers	1996-1999,2003,2005	38
SE-Sk1	60.13	17.92	ENF	Temperate-Continental with hot or warm summers	2005	----
SE-Sk2	60.13	17.84	ENF	Temperate-Continental with hot or warm summers	2004-2005	----
SK-Tat	49.12	20.16	ENF	Temperate-Continental with hot or warm summers	2005	----
UK-AMo	55.79	-3.24	WET	Temperate	2005	----
UK-EBu	55.87	-3.21	GRA	Temperate	2004, 2006	39
UK-ESa	55.91	-2.86	CRO	Temperate	2003-2005	40
UK-Gri	56.61	-3.80	ENF	Temperate	1997-1998, 2000-2002, 2005-2006	41

UK-Ham	51.15	-0.86	DBF	Temperate	2004-2005	----
UK-PL3	51.45	-1.27	DBF	Temperate	2005-2006	----
UK-Tad	51.21	-2.83	GRA	Temperate	2001	----
US-Bar	44.06	-71.29	DBF	Temperate-Continental with hot or warm summers	2004-2005	42
US-Bkg	44.35	-96.84	GRA	Temperate-Continental with hot or warm summers	2004-2006	43
US-Bo1	40.01	-88.29	CRO	Temperate-Continental with hot or warm summers	1996-2007	22
US-Ha1	42.54	-72.17	DBF	Temperate-Continental with hot or warm summers	1991-1998, 2004-2005	44
US-Ho1	45.2	-68.74	ENF	Temperate-Continental with hot or warm summers	1996-2004	45
US-Ho2	45.21	-68.75	ENF	Temperate-Continental with hot or warm summers	1999-2004	46
US-Los	46.08	-89.98	CSH	Temperate-Continental with hot or warm summers	2001-2005	47
US-Ne1	41.17	-96.48	CRO	Temperate-Continental with hot or warm	2001-2005	48

				summers		
US-Ne2	41.16	-96.47	CRO	Temperate-Continental with hot or warm summers	2001-2005	48
US-Ne3	41.18	-96.44	CRO	Temperate-Continental with hot or warm summers	2001-2005	48
US-Oho	41.55	-83.84	DBF	Temperate-Continental with hot or warm summers	2004-2005	49
US-PFa	45.95	-90.27	MF	Temperate-Continental with hot or warm summers	1996-2000,2003	50
US-Syv	46.24	-89.35	MF	Temperate-Continental with hot or warm summers	2002-2006	51
US-UMB	45.56	-84.71	DBF	Temperate-Continental with hot or warm summers	1999-2003	52
US-WCr	45.81	-90.08	DBF	Temperate-Continental with hot or warm summers	1999-2006	53
US-Wi0	46.62	-91.08	ENF	Temperate-Continental with hot or warm summers	2002	----
US-Wi1	46.73	-91.23	DBF	Temperate-Continental with hot or warm summers	2003	----

US-Wi2	46.69	-91.15	ENF	Temperate-Continental with hot or warm summers	2003	----
US-Wi4	46.74	-91.17	ENF	Temperate-Continental with hot or warm summers	2002-2005	----
US-Wi5	46.65	-91.09	ENF	Temperate-Continental with hot or warm summers	2004	----
US-Wi6	46.62	-91.30	OSH	Temperate-Continental with hot or warm summers	2002	----
US-Wi7	46.65	-91.07	OSH	Temperate-Continental with hot or warm summers	2005	----
US-Wi8	46.72	-91.25	DBF	Temperate-Continental with hot or warm summers	2002	----
US-Wi9	46.62	-91.08	ENF	Temperate-Continental with hot or warm summers	2004-2005	----

[1] Positive value indicates north latitude.

[2] Negative value indicates west longitude.

Different plant functional types: CRO, croplands; CSH, closed shrublands; GRA, grasslands; MF, mixed forests; WET, permanent wetlands; DBF, deciduous broadleaf forests; ENF, evergreen needleleaf forests; OSH, open shrublands.

SUPPORTING REFERENCES

1. Wohlfahrt, G. *et al*. Seasonal and inter-annual variability of the net ecosystem CO_2 exchange of a temperate mountain grassland: Effects of weather and management. *J. Geophys. Res-Atmos.* **113**, D08110 (2008).
2. Gielen, B. *et al*. A. Decadal water balance of a temperate Scots pine forest (Pinus sylvestris L.) based on measurements and modelling. *Biogeosciences* **7**, 1247-1261 (2010).
3. Moureaux, C., Debacq, A., Bodson, B., Heinesch, B. & Aubinet, M. Annual net ecosystem carbon exchange by a sugar beet crop. *Agr. Forest Meteorol.* **139**, 25-39 (2006).
4. Aubinet, M. *et al*. Long term carbon dioxide exchange above a mixed forest in the Belgian Ardennes. *Agr. Forest Meteorol.* **108**, 293-315 (2001).
5. Dunn, A. L., Barford, C. C., Wofsy, S. C., Goulden, M. L. & Daube, B. C. A long-term record of carbon exchange in a boreal black spruce forest: means, responses to interannual variability, and decadal trends. *Glob. Chang. Biol.* **13**, 577-590 (2007).
6. Lafleur, P. M., Roulet, N. T., Bubier, J. L., Frolking, S. & Moore, T. R. Interannual variability in the peatland-atmosphere carbon dioxide exchange at an ombrotrophic bog. *Global Biogeochem. Cy.* **17**, 1036 (2003).
7. Goulden, M. L., Miller, S. D. & da Rocha, H. R. Nocturnal cold air drainage and pooling in a tropical forest. *J. Geophys. Res-Atmos.* **111**, D08S04 (2006).
8. Giasson, M.-A., Coursolle, C. & Margolis, H. A. Ecosystem-level CO_2 fluxes from a boreal cutover in eastern Canada before and after scarification. *Agr. Forest Meteorol.* **140**, 23-40 (2006).
9. Bergeron, O. *et al.* Comparison of carbon dioxide fluxes over three boreal

black spruce forests in Canada. *Glob. Chang. Biol.* **13**, 89-107 (2007).

10. Zha, T. *et al.* Carbon sequestration in boreal jack pine stands following harvesting. *Glob. Chang. Biol.* **15**, 1475-1487 (2009).

11. Ammann, C., Flechard, C. R., Leifeld, J., Neftel, A. & Fuhrer, J. The carbon budget of newly established temperate grassland depends on management intensity. *Agr. Ecosyst. Environ.* **121**, 5-20 (2007).

12. Reichstein, M. *et al.* Modeling temporal and large-scale spatial variability of soil respiration from soil water availability, temperature and vegetation productivity indices. *Global Biogeochem. Cy.* **17**, 1104 (2003).

13. Valentini, R. *et al.* Respiration as the main determinant of carbon balance in European forests. *Nature* **404**, 861-865 (2000).

14. Anthoni, P. M. *et al.* Forest and agricultural land-use-dependent CO_2 exchange in Thuringia, Germany. *Glob. Chang. Biol.* **10**, 2005-2019 (2004).

15. Gilmanov, T. G., *et al.* Partitioning European grassland net ecosystem CO_2 exchange into gross primary productivity and ecosystem respiration using light response function analysis. *Agr. Ecosyst. Environ.* **121**, 93-120 (2007).

16. Knohl, A., Schulze, E.-D., Kolle, O. & Buchmann, N. Large carbon uptake by an unmanaged 250-year-old deciduous forest in Central Germany. *Agr. Forest Meteorol.* **118**, 151-167 (2003).

17. Chen, B. *et al.* A data-model fusion approach for upscaling gross ecosystem productivity to the landscape scale based on remote sensing and flux footprint modelling. *Biogeosciences* **7**, 2943-2958 (2010).

18. Don, A., Rebmann, C., Kolle, O., Scherer-Lorenzen, M. & Schulze, E.-D. Impact of afforestation-associated management changes on the carbon balance of grassland. *Glob. Chang. Biol.* **15**, 1990-2002 (2009).

19. Arain, M. A. & Restrepo-Coupe, N. Net ecosystem production in a temperate pine plantation in southeastern Canada. *Agr. Forest Meteorol.* **128**, 223-241 (2005).

20. Rebmann, C. *et al.* Treatment and assessment of the CO_2-exchange at a complex forest site in Thuringia, Germany. *Agr. Forest Meteorol.* **150**, 684-691 (2010).

21. Pilegaard, K. *et al*. Field measurements of atmosphere-biosphere interactions in a Danish beech forest. *Boreal Environ. Res.* **8**, 315-334 (2003).

22. Migliavacca, M. *et al.* Semiempirical modeling of abiotic and biotic factors controlling ecosystem respiration across eddy covariance sites. *Glob. Chang. Biol.* **17**, 390-409 (2011).

23. Suni, T. *et al.* Long-term measurements of surface fluxes above a Scots pine forest in Hyytiala, southern Finland, 1996-2001. *Boreal Environ. Res.* **8**, 287-302 (2003).

24. Granier, A. *et al.* The carbon balance of a young Beech forest. *Funct. Ecol.* **14**, 312-325 (2000).

25. Berbigier, P., Bonnefond, J.-M. & Mellmann, P. CO_2 and water vapour fluxes for 2 years above Euroflux forest site. *Agr. Forest Meteorol.* **108**, 183-197 (2001).

26. Pintér, K. *et al.* Interannual variability of grasslands' carbon balance depends on soil type. *Community Ecol.* **9**, 43-48 (2008).

27. Jaksic, V. *et al.* Net ecosystem exchange of grassland in contrasting wet and dry years. *Agr. Forest Meteorol.* **139**, 323-334 (2006).

28. Marcolla, B., Pitacco, A. & Cescatti, A. Canopy Architecture and Turbulence Structure in a Coniferous Forest. *Bound-Lay. Meteorol.* **108**, 39-59 (2003).

29. Migliavacca, M. *et al.* Modeling gross primary production of Agro-Forestry ecosystems by assimilation of satellite-derived information in a process-based model. *Sensors* **9**, 922-942 (2009).

30. Montagnani, L. *et al.* A new mass conservation approach to the study of CO_2 advection in an alpine forest. *J. Geophys. Res-Atmos.* **114**, D07306 (2009).

31. Jacobs, C. M. J. *et al.* Variability of annual CO_2 exchange from Dutch Grasslands. *Biogeosci. Discuss.*, European Geosciences Union **4**, 1499-1534 (2007).

32. Dolman, A. J., Moors, E. J. & Elbers, J. A. The carbon uptake of a mid latitude pine forest growing on sandy soil. *Agr. Forest Meteorol.* **111**, 157-170 (2002).

33. Chojnicki, B. H., Urbaniak, M., Józefczyk, D., Augustin, J. & Olejnik, J. Measurements of gas and heat fluxes at Rzecin wetland. *Wetlands: Monitoring, Modeling and Management (eds Okruszko T, Maltby E, Szatylowicz J, Miroslaw-Swiatek D, Kotowski W)*, 125-131 (2007).

34. Kurbatova, J., Li, C., Varlagin, A., Xiao, X. & Vygodskaya, N. Modeling carbon dynamics in two adjacent spruce forests with different soil conditions in Russia. *Biogeosciences* **5**, 969-980 (2008).

35. Chevallier, F., Viovy, N., Reichstein, M. & Ciais, P. On the assignment of prior errors in Bayesian inversions of CO_2 surface fluxes. *Geophys. Res. Lett.* **33**, L13802 (2006).

36. Lund, M., Lindroth, A., Christensen, T. R. & StrÖM, L. Annual CO_2 balance of a temperate bog. *Tellus B* **59**, 804-811 (2007).

37. Lindroth, A., Klemedtsson, L., Grelle, A., Weslien, P. & Langvall, O. Measurement of net ecosystem exchange, productivity and respiration in three

spruce forests in Sweden shows unexpectedly large soil carbon losses. *Biogeochemistry* **89**, 43-60 (2008).

38. Lagergren, F. *et al.* Biophysical controls on CO_2 fluxes of three Northern forests based on long-term eddy covariance data. *Tellus B* **60**, 143-152 (2008).

39. Yi, C., *et al.* Climate control of terrestrial carbon exchange across biomes and continents. *Environ. Res. Lett.* **5**, 034007 (2010).

40. Groenendijk, M. *et al.* Assessing parameter variability in a photosynthesis model within and between plant functional types using global FLUXNET eddy covariance data. *Agr. Forest Meteorol.* **151**, 22-38 (2011).

41. Rebmann, C. *et al.* Quality analysis applied on eddy covariance measurements at complex forest sites using footprint modelling. *Theor. Appl. Climatol.* **80**, 121-141 (2005).

42. Jenkins, J. P. *et al.* Refining light-use efficiency calculations for a deciduous forest canopy using simultaneous tower-based carbon flux and radiometric measurements. *Agr. Forest Meteorol.* **143**, 64-79 (2007).

43. Gilmanov, T. G. *et al.* Integration of CO_2 flux and remotely-sensed data for primary production and ecosystem respiration analyses in the Northern Great Plains: potential for quantitative spatial extrapolation. *Global Ecol. Biogeogr.* **14**, 271-292 (2005).

44. Urbanski, S. *et al.* Factors controlling CO_2 exchange on timescales from hourly to decadal at Harvard Forest. *J. Geophys. Res-Biogeosci.* **112**, G02020 (2007).

45. Hollinger, D. Y. *et al.* Spatial and temporal variability in forest–atmosphere CO_2 exchange. *Glob. Chang. Biol.* **10**, 1689-1706 (2004).

46. Oren, R. A. M. *et al.* Estimating the uncertainty in annual net ecosystem

carbon exchange: spatial variation in turbulent fluxes and sampling errors in eddy-covariance measurements. *Glob. Chang. Biol.* **12**, 883-896 (2006).

47. Sulman, B. N., Desai, A. R., Cook, B. D., Saliendra, N. & Mackay, D. S. Contrasting carbon dioxide fluxes between a drying shrub wetland in Northern Wisconsin, USA, and nearby forests. *Biogeosciences* **6**, 1115-1126 (2009).
48. Richardson, A. D. *et al.* A multi-site analysis of random error in tower-based measurements of carbon and energy fluxes. *Agr. Forest Meteorol.* **136**, 1-18 (2006).
49. DeForest, J. L. *et al.* Phenophases alter the soil respiration–temperature relationship in an oak-dominated forest. *Int. J. Biometeorol.* **51**, 135-144 (2006).
50. Davis, K. J. *et al.* The annual cycles of CO_2 and H_2O exchange over a northern mixed forest as observed from a very tall tower. *Glob. Chang. Biol.* **9**, 1278-1293 (2003).
51. Desai, A. R., Bolstad, P. V., Cook, B. D., Davis, K. J. & Carey, E. V. Comparing net ecosystem exchange of carbon dioxide between an old-growth and mature forest in the upper Midwest, USA. *Agr. Forest Meteorol.* **128**, 33-55 (2005).
52. Gough, C. M., Vogel, C. S., Schmid, H. P., Su, H. B. & Curtis, P. S. Multi-year convergence of biometric and meteorological estimates of forest carbon storage. *Agr. Forest Meteorol.* **148**, 158-170 (2008).
53. Cook, B. D. *et al.* Carbon exchange and venting anomalies in an upland deciduous forest in northern Wisconsin, USA. *Agr. Forest Meteorol.* **126**, 271-295 (2004).
